# Admission Endothelial Activation and Stress Index in Acute Myocardial Infarction: Incremental Value Beyond the GRACE Score and a Test of the Index Weights

**DOI:** 10.3390/jcm15197703

**Published:** 2026-10-05

**Authors:** Kader Eliz Şahin, Ahmet Berk Duman, Şadiye Nur Dalgıç, Denizhan Karaçimen, Yalçın Dalgıç, Metin Çağdaş, Sedat Avcı, Ayşe Hoşoğlu

**Affiliations:** 1 Department of Cardiology, University of Health Sciences, Kocaeli City Hospital, 41060 İzmit, Türkiye; dumanahmetberk@gmail.com (A.B.D.);; 2 Independent Researcher, 06000 Ankara, Türkiye

**Keywords:** Endothelial Activation and Stress Index, myocardial infarction, hospital mortality, GRACE score, risk assessment, prediction model validation

## Abstract

**Background/Objectives:** The Endothelial Activation and Stress Index (EASIX), calculated from lactate dehydrogenase, creatinine and platelet count, has been linked to mortality in cardiovascular cohorts. We examined whether admission EASIX adds prognostic information to the Global Registry of Acute Coronary Events (GRACE) score for in-hospital mortality in acute myocardial infarction (AMI), and whether its fixed weights are supported. **Methods:** We retrospectively studied consecutive adults with AMI undergoing coronary angiography; patients with an estimated glomerular filtration rate below 30 mL/min/1.73 m^2^ were excluded. EASIX was modelled on the log2 scale. Incremental value was assessed against a GRACE model refitted in this cohort and against the published GRACE risk after recalibration, by discrimination, calibration, decision curves and bootstrap validation. **Results:** In-hospital death occurred in 225 of 4777 patients (4.7%). Each doubling of EASIX was associated with mortality after adjustment for the GRACE score (odds ratio 2.386, 95% confidence interval 2.053–2.774) and after clinical adjustment (2.363, 1.974–2.828). Adding EASIX raised the area under the curve from 0.855 to 0.890 (optimism-corrected increment 0.034, 95% confidence interval 0.025–0.052) and improved the Brier score and net benefit. Added to the recalibrated published GRACE risk, EASIX raised the area under the curve from 0.856 to 0.881 (corrected increment 0.024, 0.012–0.040). Findings were consistent in both infarct types. Freely estimated weights differed from the fixed weights but added little discrimination. **Conclusions:** Admission EASIX was independently associated with in-hospital mortality in AMI and added discrimination and net benefit to both a refitted and the published GRACE risk. External validation is required before clinical use.

## 1. Introduction

Acute myocardial infarction remains a leading cause of hospital death despite timely reperfusion and guideline-directed therapy, and early risk stratification is central to decisions about monitoring intensity and the timing of invasive management [1]. Endothelial dysfunction is a shared mechanism across acute coronary syndromes, linking plaque instability, microvascular obstruction and inflammatory activation, and endothelial function is impaired in these patients [2]. Direct assessment is impractical at the bedside, so composite indices built from routine admission laboratory values have attracted interest as inexpensive, immediately available markers that could refine risk beyond established clinical scores.

The Endothelial Activation and Stress Index (EASIX) is one such composite, defined as lactate dehydrogenase (LDH) multiplied by creatinine and divided by platelet count. It was derived in acute graft-versus-host disease [3] and predicts mortality after allogeneic stem cell transplantation [4]; its link to the endothelium is correlative, resting on parallel rises in vascular cell adhesion molecule-1, tumour necrosis factor receptor 1 and von Willebrand factor antigen in transplant recipients [5]. In cardiovascular cohorts EASIX has been associated with mortality after cardiac catheterisation [6] and in intensive care [7,8], with contrast-induced nephropathy, in-hospital mortality and the no-reflow phenomenon in ST-elevation infarction [9,10,11], with one-year mortality in non-ST-elevation infarction [12], and with outcomes in acute coronary syndrome broadly [13] and in a nationally representative cohort [14]. Recent evidence now includes a dual-cohort study in acute myocardial infarction (AMI) evaluating the incremental performance of EASIX beyond the Global Registry of Acute Coronary Events (GRACE) score with discrimination, calibration, prediction error and decision curve analyses [15]. Two questions remain insufficiently addressed. First, the incremental value of EASIX against the original published in-hospital GRACE risk after recalibration, including performance within each infarct type, remains uncertain. Second, the component weights inherited from the haematological derivation of the index [3] have not been tested against freely estimated weights and component-wise models in an AMI population.

We therefore studied consecutive patients with AMI undergoing invasive coronary angiography at a tertiary centre. The primary aim was to determine whether admission EASIX adds prognostic information for in-hospital all-cause mortality to the GRACE score [16], assessed both against a GRACE model refitted in this cohort and against the published GRACE risk. The secondary aims were to test whether the fixed component weights of the index are supported by these data and whether its contribution rests on the composite or on its individual components.

## 2. Materials and Methods

### 2.1. Study Design and Population

This was a retrospective, single-centre cohort study. Reporting follows the Strengthening the Reporting of Observational Studies in Epidemiology (STROBE) statement for cohort studies, and the completed checklist accompanies this submission. Because the analysis evaluates an existing index and its incremental value over an established risk score, the relevant items of the TRIPOD statement were also followed [17].

**Figure 1:**
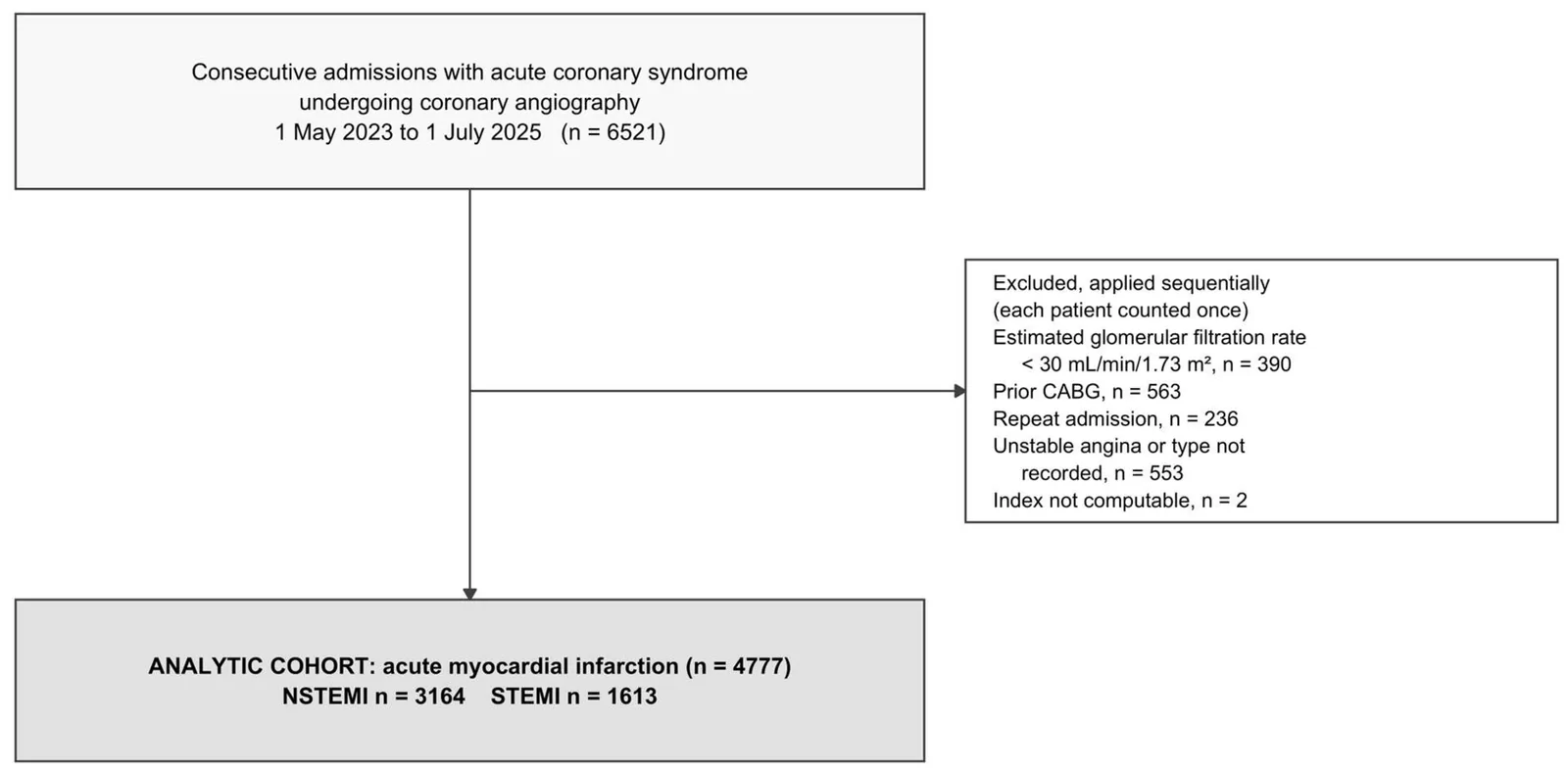
Patient selection flow. Consecutive admissions with an acute coronary syndrome undergoing coronary angiography between 1 May 2023 and 1 July 2025. Exclusion criteria were applied sequentially, and each patient is counted once, against the first criterion met. CABG, coronary artery bypass grafting; NSTEMI, non-ST-elevation myocardial infarction; STEMI, ST-elevation myocardial infarction.

Between May 2023 and July 2025, 6521 consecutive adult patients with acute coronary syndrome undergoing coronary angiography at Kocaeli City Hospital were screened. Patients with an estimated glomerular filtration rate (eGFR) < 30 mL/min/1.73 m^2^ (*n* = 390), prior coronary artery bypass grafting (CABG) (*n* = 563), repeat admissions (*n* = 236), unstable angina or unrecorded syndrome type (*n* = 553), and an admission index not computable because of an invalid baseline creatinine value (*n* = 2) were excluded. The eGFR threshold was a cohort-construction criterion rather than a cutoff selected from the observed outcomes: the intended scope was patients without advanced kidney disease, because chronic renal dysfunction directly affects one of the three EASIX components and may alter both the performance and the relative component weights of the index. These exclusions, the eGFR threshold in particular, truncate the admission creatinine distribution and remove patients at high baseline risk of death, so the performance of EASIX reported here is conditional on these eligibility criteria. The final analytic cohort comprised 4777 AMI patients, 3164 with non-ST-elevation myocardial infarction (NSTEMI) and 1613 with ST-elevation myocardial infarction (STEMI) (Figure 1).

### 2.2. Ethics

The study was approved by the Scientific Research Ethics Committee of Kocaeli City Hospital (protocol number 2025-190, approved 8 January 2026) and conducted in accordance with the Declaration of Helsinki. The requirement for individual informed consent was waived because of the retrospective design and the use of de-identified routinely collected data. Patient identifiers were removed before analysis.

### 2.3. Outcomes

The primary outcome was in-hospital all-cause mortality during the index admission, including deaths after in-hospital transfer to cardiac surgery. The secondary outcome was contrast-associated acute kidney injury (CA-AKI), defined by the Kidney Disease: Improving Global Outcomes creatinine criteria as a rise in serum creatinine of at least 0.3 mg/dL or to at least 1.5 times the admission value. Creatinine measurements were not time-stamped, so the peak in-hospital value was used and the 48 h and 7-day windows of the definition could not be verified; CA-AKI was assessed in patients with at least one creatinine measurement after admission. Follow-up beyond discharge was not systematically ascertained and no censoring date was available, so no time-to-event analysis was performed.

### 2.4. Data Collection and GRACE Score

Demographic characteristics, comorbidities, admission vital signs, echocardiographic left ventricular ejection fraction (LVEF), admission laboratory values, the revascularisation strategy of the index admission (medical therapy, percutaneous coronary intervention, or heart-team decision or CABG) and the final thrombolysis in myocardial infarction (TIMI) flow grade were extracted from the institutional electronic record. Laboratory values were those obtained at admission, before coronary angiography. eGFR values were those reported by the hospital laboratory, which uses the CKD-EPI 2009 creatinine equation [18].

The risk score used throughout is the original numerical GRACE 1.0 score for in-hospital death described by Granger et al. [16], not the subsequently developed GRACE 2.0 or GRACE 3.0 models; it comprises eight components: age, heart rate, systolic blood pressure, serum creatinine, Killip class, cardiac arrest at admission, ST-segment deviation and elevated cardiac biomarkers. Points were computed for every patient from the recorded admission values with the published nomogram. Elevated cardiac biomarkers were defined from source-record documentation of an admission high-sensitivity troponin T above the 99th-percentile upper reference limit of 14 ng/L. A numeric high-sensitivity troponin T value was not retained in the analytic file for 16 patients, but their documented positive cardiac-marker status was available and was used for GRACE scoring. ST-segment depression was not recorded, so ST-deviation points were assigned to ST-elevation infarction. The published probability of in-hospital death was obtained from the points-to-probability table of the same nomogram [16].

### 2.5. EASIX and Its Components

EASIX was recalculated for every patient from the admission values as

*EASIX* = *LDH* (U/L) × *creatinine* (mg/dL)/*platelet count* (×10^9^/L)

and, because the distribution is right-skewed, was analysed after base-2 logarithmic transformation. The pre-existing index field in the source database was not used.

The exponents of the three components were also estimated freely, giving a freely weighted index

*LDH* × *creatinine^a^*/*platelet count^b^*

A logistic model for in-hospital death was fitted with log2 LDH, log2 creatinine and log2 platelet count as three free terms, and the three coefficients were divided by the LDH coefficient, so that a and b are expressed relative to LDH and no exponent was fixed a priori. Uncertainty in the re-derived weights was quantified by bootstrap with 500 resamples and is reported as percentile 95% confidence intervals. This is a test of the weights implicit in EASIX, not the proposal of a replacement index. Because the GRACE score carries creatinine only in categorised form, part of any incremental value of EASIX could reflect re-entry of continuous creatinine rather than the composite itself. To separate the two, with models adding each component alone, each pair of components and all three components of the GRACE score were compared with the model adding EASIX, and the independent contribution of each component was tested by removing it from the three-component model.

### 2.6. Statistical Analysis

Continuous variables are summarised as median and interquartile range and compared with the Kruskal–Wallis test; categorical variables are summarised as counts with percentages and compared with the chi-square test. Trend in mortality across EASIX tertiles was tested by entering the tertile rank as a single linear term in a logistic model. Tertiles are descriptive; the primary exposure is the continuous index, modelled on the log2 scale so that the odds ratio corresponds to a doubling of EASIX. Linearity was assessed with four-degrees-of-freedom restricted cubic splines, for the index on the log2 scale and for the GRACE score on its points scale.

Logistic regression models were fitted for the primary outcome: unadjusted (Model 1); adjusted for the GRACE score entered as a restricted cubic spline with four degrees of freedom (interior knots at 120, 139 and 160; boundary knots at 51 and 332), the primary GRACE-adjusted specification because linearity was rejected (Model 2); the same adjustment with the score as a linear term, the specification used in the existing literature (Model 2a); and adjusted for age, sex, Killip class, infarct type and eGFR, the covariates with complete data in the analytic cohort (Model 3). Every adjusted odds ratio quoted for EASIX is from the spline specification unless the linear term is named. Because Killip class, age, systolic blood pressure, heart rate and creatinine are components of the GRACE score, the score and its components were never entered into the same model.

Discrimination was quantified by the area under the receiver operating characteristic curve (AUC) with DeLong confidence intervals; models were compared with the DeLong test on identical records [19]. Calibration was assessed by the Hosmer–Lemeshow test and the Brier score, and displayed by decile of predicted risk together with a loess-smoothed calibration curve. Models refitted in this cohort have calibration-in-the-large and a calibration slope of 1 by construction, so their calibration assessment concerns the shape of the risk relationship. The published GRACE probabilities were therefore also evaluated directly, by the observed-to-expected ratio, calibration-in-the-large, calibration slope, AUC and Brier score; their linear predictor was then recalibrated by re-estimating an intercept and a slope, and EASIX was added to the recalibrated model. Incremental value was assessed by likelihood ratio test, the integrated discrimination improvement (IDI) [20] and decision curve analysis [21], with threshold probabilities of 1% to 30%; because the DeLong test is conservative for nested models [22], these measures were read together with the change in AUC. This range spans escalation decisions of low-to-moderate burden in the infarct setting, from intensified monitoring to coronary-care admission or earlier invasive management; no single intervention was prespecified, so the curves screen for decision-analytic value rather than evaluate a named treatment policy. Base and augmented models were internally validated together by bootstrap (500 identical resamples, with knot placement and the choice between linear and spline entry of the GRACE score repeated in each); optimism-corrected AUC, AUC difference, Brier score, IDI and net benefit are reported, with percentile 95% confidence intervals for the AUC difference and the IDI, and the bootstrap calibration slope and intercept.

Sensitivity analyses of the GRACE-adjusted estimate were as follows: repetition after 1% winsorisation of the three EASIX components; substitution of the freely weighted index for EASIX; a restricted cubic spline for log2 EASIX; exclusion of deaths after transfer to cardiac surgery; exclusion of patients referred to the heart team or to CABG; assignment of ST-deviation points to every NSTEMI patient, as an upper bound for the unrecorded ST-segment depression; addition of the revascularisation strategy and the final TIMI flow grade, which are post-admission variables and were therefore not part of the primary admission-risk model; and addition of log2 high-sensitivity troponin T and of LVEF in the patients in whom they were recorded. All models were also fitted separately within each infarct type, and effect modification was tested with an interaction term.

Sample size was fixed by the available cohort; this is an evaluation of an existing index rather than the development of a new prediction model, and no post hoc power calculation was performed. Parameter burden, outcome frequency and the resulting precision are reported alongside each model. The primary models are fitted on all 4777 patients in the analytic cohort, so no primary estimate depends on the handling of missing data; the troponin and LVEF sensitivity analyses are complete-case.

One outcome was treated as primary, in-hospital all-cause mortality, and no correction for multiplicity was applied to it. CA-AKI is a secondary outcome and is reported descriptively only, as tertile event rates and an AUC with no adjusted model, because admission creatinine is a component of the index and the outcome is defined as a rise from that same value. Subgroup and interaction analyses, the tertile cutpoints and the re-weighting analysis are exploratory and were not corrected for multiplicity.

Analyses were performed in R version 4.5.2 using haven version 2.5.5, pROC version 1.19.1, splines (base R 4.5.2) and ggplot2 version 4.0.3. The random seed was 20260907.

## 3. Results

Among the 4777 patients in the analytic cohort, in-hospital death occurred in 225 (4.7%), 13 of them after transfer to cardiac surgery. Of the 212 deaths with a recorded day, the median time to death was 2.38 days (interquartile range 0.59 to 9.57); 38.2% occurred within 24 h and 47.2% within 48 h. CA-AKI occurred in 646 of the 4761 patients with a creatinine measurement after admission (13.6%), dialysis was required in 73 patients (1.5%) and stent thrombosis occurred in 22 (0.5%). Median length of stay was 2.23 days (interquartile range 1.62 to 3.91), and 91.1% of survivors were discharged before day 7.

Median age was 62 years (interquartile range 53 to 71), 3326 patients (69.6%) were men and 797 (16.7%) presented in Killip class II or above. Median LVEF, recorded in 3416 patients, was 53% (43 to 60), and the median GRACE score was 139 (120 to 160). Median EASIX was 1.22 (0.83 to 1.87). Medical therapy was the strategy in 1232 patients (25.8%), percutaneous coronary intervention in 2968 (62.1%), and a heart-team decision or CABG in 577 (12.1%).

Baseline characteristics by EASIX tertile are shown in Table 1. Patients in higher tertiles were older (median 59, 62 and 64 years), more often men (60.4%, 72.0% and 76.5%), more often presented with ST-elevation infarction (25.0%, 32.4% and 43.9%) and more often underwent percutaneous coronary intervention (56.1%, 63.1% and 67.3%). LVEF fell across tertiles (60%, 55% and 50%) and eGFR declined (94, 84 and 69 mL/min/1.73 m^2^). By construction the components diverged: LDH rose from 236 to 308 to 475 U/L, creatinine from 0.79 to 0.92 to 1.08 mg/dL, and platelet count fell from 283 to 242 to 215 ×10^9^/L. The proportion presenting in Killip class II or above did not differ across tertiles (16.0%, 17.9% and 16.1%; *p* = 0.278).

In-hospital mortality rose across EASIX tertiles from 14 of 1593 (0.9%) in the lowest, to 48 of 1592 (3.0%) in the middle, to 163 of 1592 (10.2%) in the highest tertile (*p* for trend <0.001; Supplementary Table S1).

Modelled continuously, each doubling of EASIX was associated with an unadjusted odds ratio for in-hospital death of 3.146 (95% confidence interval 2.738 to 3.614). The association persisted after adjustment for the GRACE score entered as a restricted cubic spline, the primary specification (odds ratio 2.386, 2.053 to 2.774; Model 2), and after adjustment for age, sex, Killip class, infarct type and eGFR (odds ratio 2.363, 1.974 to 2.828; Model 3), in which age, sex, Killip class, infarct type and eGFR were also independently associated with mortality (Table 2). With the score entered instead as a linear term, the corresponding odds ratio was 2.554 (2.202 to 2.963; Model 2a); the spline model fitted better (Akaike information criterion 1293.7 against 1360.3).

**Figure 2:**
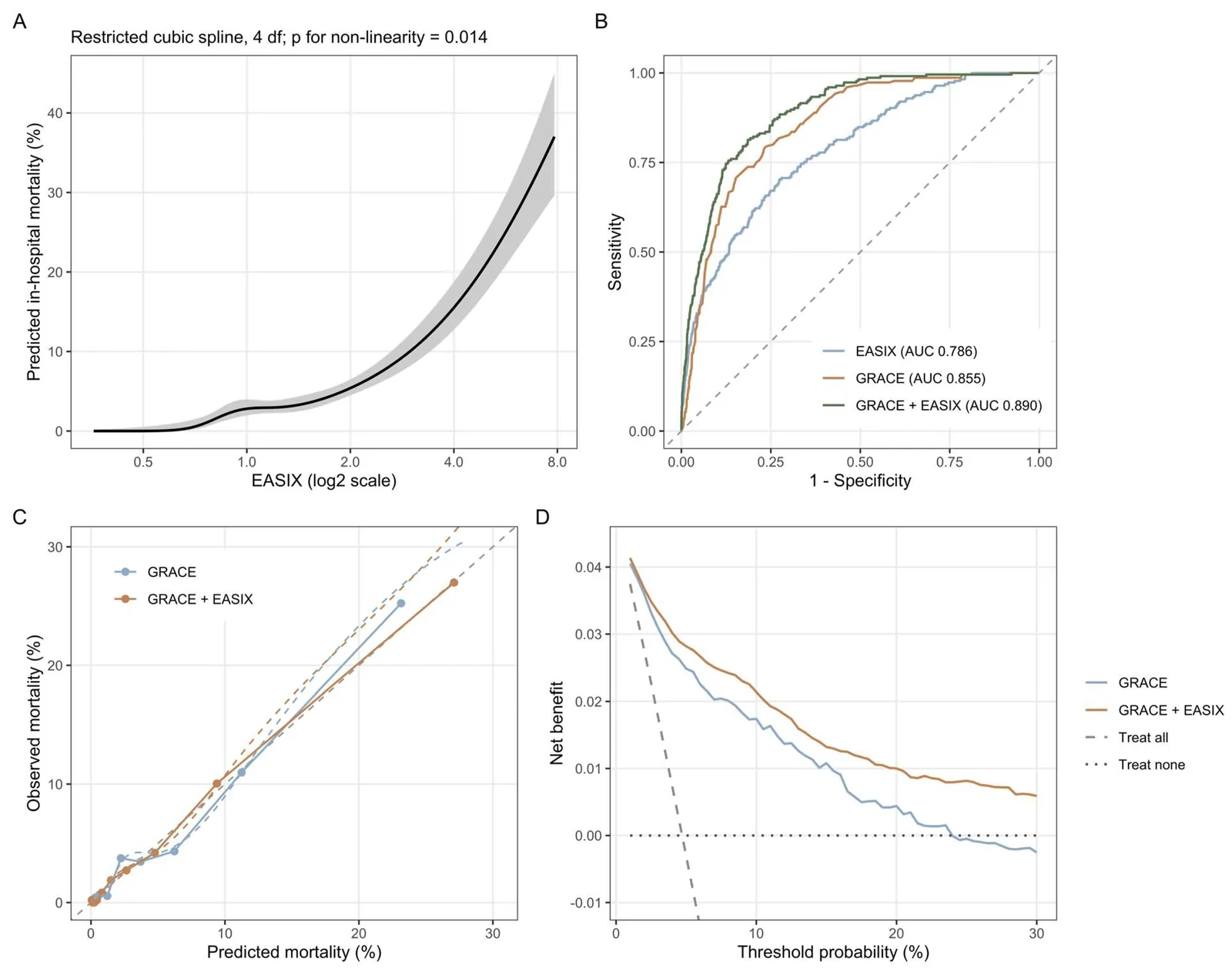
Model performance for in-hospital mortality (*n* = 4777, 225 deaths). (**A**) Restricted cubic spline with four degrees of freedom for predicted mortality against EASIX on the log2 scale; shaded band is the 95% confidence interval. (**B**) Receiver operating characteristic curves for the index, for a model containing the GRACE score as a spline, and for the two combined; the diagonal denotes no discrimination (area under the curve 0.50). (**C**) Calibration by decile of predicted risk (points and solid lines) with loess-smoothed calibration curves (dashed); the diagonal denotes perfect calibration. (**D**) Decision curve analysis; net benefit of the GRACE model, of GRACE with EASIX added, and of treating all or no patients, against threshold probability. EASIX, Endothelial Activation and Stress Index; GRACE, Global Registry of Acute Coronary Events. The dashed line denotes the treat-all strategy and the dotted horizontal line denotes the treat-none strategy.

The relationship on the log2 scale departed modestly from linearity (restricted cubic spline with four degrees of freedom against a linear term, *p* for non-linearity = 0.014; Figure 2A), with risk rising most steeply at the upper end of the distribution. In the GRACE-adjusted model a spline for log2 EASIX improved fit marginally (likelihood ratio *p* = 0.024) without changing discrimination (AUC 0.890 with the linear term and 0.893 with the spline), so the linear term, which yields a single interpretable odds ratio, was retained. Categorising the index into tertiles reduced discrimination from an AUC of 0.786 to 0.732, so tertiles are reported for description only.

Sensitivity analyses of Model 2 were consistent with it (Supplementary Table S2). After 1% winsorisation of all three components the odds ratio per doubling was 2.466 (2.104 to 2.891), and the AUC of the winsorised index was unchanged at 0.786. Substituting the freely weighted index for EASIX in the same model gave an odds ratio of 2.300 (1.993 to 2.654).

Each component considered separately discriminated less well than the composite: the AUC was 0.737 (0.702 to 0.772) for LDH, 0.727 (0.693 to 0.761) for creatinine and 0.550 (0.506 to 0.594) for platelet count, against 0.786 (0.756 to 0.816) for EASIX; the paired DeLong test against EASIX gave *p* < 0.001 for each component.

The GRACE points scale was strongly non-linear on the log-odds scale (spline against linear term, *p* < 0.001). The AUC for in-hospital mortality was 0.786 (0.756 to 0.816) for EASIX alone and 0.855 (0.833 to 0.877) for a model containing the GRACE score as a spline (Table 3, Figure 2B). Adding EASIX raised it to 0.890 (0.871 to 0.909), with a DeLong *p* < 0.001, a likelihood ratio test *p* < 0.001 and an IDI of 0.078. With the GRACE score entered as a linear term the AUC rose from 0.856 (0.834 to 0.878) to 0.881 (0.861 to 0.900), with a DeLong *p* = 0.009 and an IDI of 0.076. Adding EASIX to the clinical model raised the AUC from 0.914 (0.899 to 0.930) to 0.929 (0.916 to 0.942), with a DeLong *p* = 0.002 and an IDI of 0.056.

Among the refitted models (Table 3), the model entering the GRACE score as a linear term showed evidence of miscalibration (Hosmer–Lemeshow *p* < 0.001), which persisted with EASIX added (*p* = 0.003). The spline model showed no evidence of miscalibration either without (*p* = 0.146) or with EASIX (*p* = 0.745), nor did EASIX alone (*p* = 0.511); the clinical model retained evidence of miscalibration without and with EASIX (*p* = 0.008 and *p* = 0.003). The loess-smoothed calibration curve of the GRACE spline model rose above the identity line at predicted risks of about 15% to 25%, indicating under-prediction at the upper end, whereas the curve of the combined model lay closer to the identity line (Figure 2C). The Brier score improved from 0.0402 for the GRACE spline model to 0.0368 with EASIX added, and from 0.0388 for the clinical model to 0.0360.

Bootstrap internal validation of both models together on identical resamples, knot placement and the choice of spline over linear entry included, left these findings essentially unchanged (Table 3). The optimism-corrected AUC was 0.855 for the GRACE spline model and 0.889 for GRACE plus EASIX; the increment of 0.036 fell to 0.034 after optimism correction (bootstrap 95% confidence interval 0.025 to 0.052). The optimism-corrected Brier scores were 0.0401 and 0.0369, and the optimism-corrected IDI was 0.077 (0.050 to 0.108). Bootstrap calibration slopes were 0.977 and 0.976, with intercepts of −0.008 for both models. The spline was preferred over linear entry by the Akaike information criterion in all 500 resamples.

By guideline GRACE category, 723 patients (15.1%) scored 108 or less, 1786 (37.4%) scored 109 to 140 and 2268 (47.5%) scored above 140, with in-hospital mortality of 0.1%, 0.6% and 9.4% (Table 4). Within the high-risk category, mortality rose across EASIX tertiles from 2.3% (13 of 571) to 6.4% (48 of 745) and 16.0% (152 of 952). In the intermediate category all 11 deaths occurred in the highest EASIX tertile (2.2%, 11 of 494), and in the low-risk category one death occurred.

Applied without refitting, the published GRACE risk predicted a mean in-hospital mortality of 5.4% against an observed 4.7% (observed-to-expected ratio 0.86; calibration-in-the-large −0.188; calibration slope 0.852), with an AUC of 0.856 (0.834 to 0.878) (Table 5). The observed-to-expected ratio was 0.80 in NSTEMI and 0.91 in STEMI. After recalibration of its intercept and slope, adding EASIX raised the AUC from 0.856 to 0.881, with an optimism-corrected increment of 0.024 (0.012 to 0.040), a DeLong *p* = 0.009, a likelihood ratio test *p* < 0.001 and an IDI of 0.076. The corrected increment was 0.025 (0.004 to 0.059) in NSTEMI and 0.023 (0.003 to 0.046) in STEMI. With ST-deviation points assigned to every NSTEMI patient, the published risk over-predicted more markedly (observed-to-expected ratio 0.58), and the corrected increment with EASIX was 0.038 (0.024 to 0.058).

Across threshold probabilities from 1% to 30%, decision curve analysis showed higher net benefit for GRACE plus EASIX than for GRACE alone at every threshold; from a threshold of 24% the net benefit of GRACE alone fell below that of treating no patient, whereas GRACE plus EASIX remained above it throughout (Figure 2D). Net benefit changed little after optimism correction: at threshold probabilities of 5%, 10%, 15% and 20%, the corrected net benefit of GRACE plus EASIX was 0.0278, 0.0208, 0.0126 and 0.0094, against 0.0246, 0.0171, 0.0104 and 0.0040 for GRACE alone (Table 3).

CA-AKI rose across EASIX tertiles from 8.7% to 10.7% to 21.4% (Table 1), and the AUC for EASIX was 0.645 (0.622 to 0.669), lower than for the primary outcome. This estimate must be read against the structural relationship between the index and the outcome, since admission creatinine is a component of EASIX and the outcome is defined by a rise from that same admission value.

Estimating the three component exponents freely given, relative to a weight of 1.000 for log2 LDH, a weight of 1.605 for log2 creatinine (bootstrap 95% confidence interval 1.213 to 2.046) and −0.626 for log2 platelet count (−0.935 to −0.333). Both intervals exclude the values implicit in EASIX, and the likelihood ratio test against the fixed-weight model was significant (chi-square 22.63, two degrees of freedom, *p* < 0.001), with the Akaike information criterion falling from 1537.5 to 1518.9. The freely weighted index gave an AUC of 0.795 (0.765 to 0.825) against 0.786 (0.756 to 0.816) for EASIX, a difference for which the DeLong test gave *p* = 0.074 (Supplementary Table S3).

**Figure 3:**
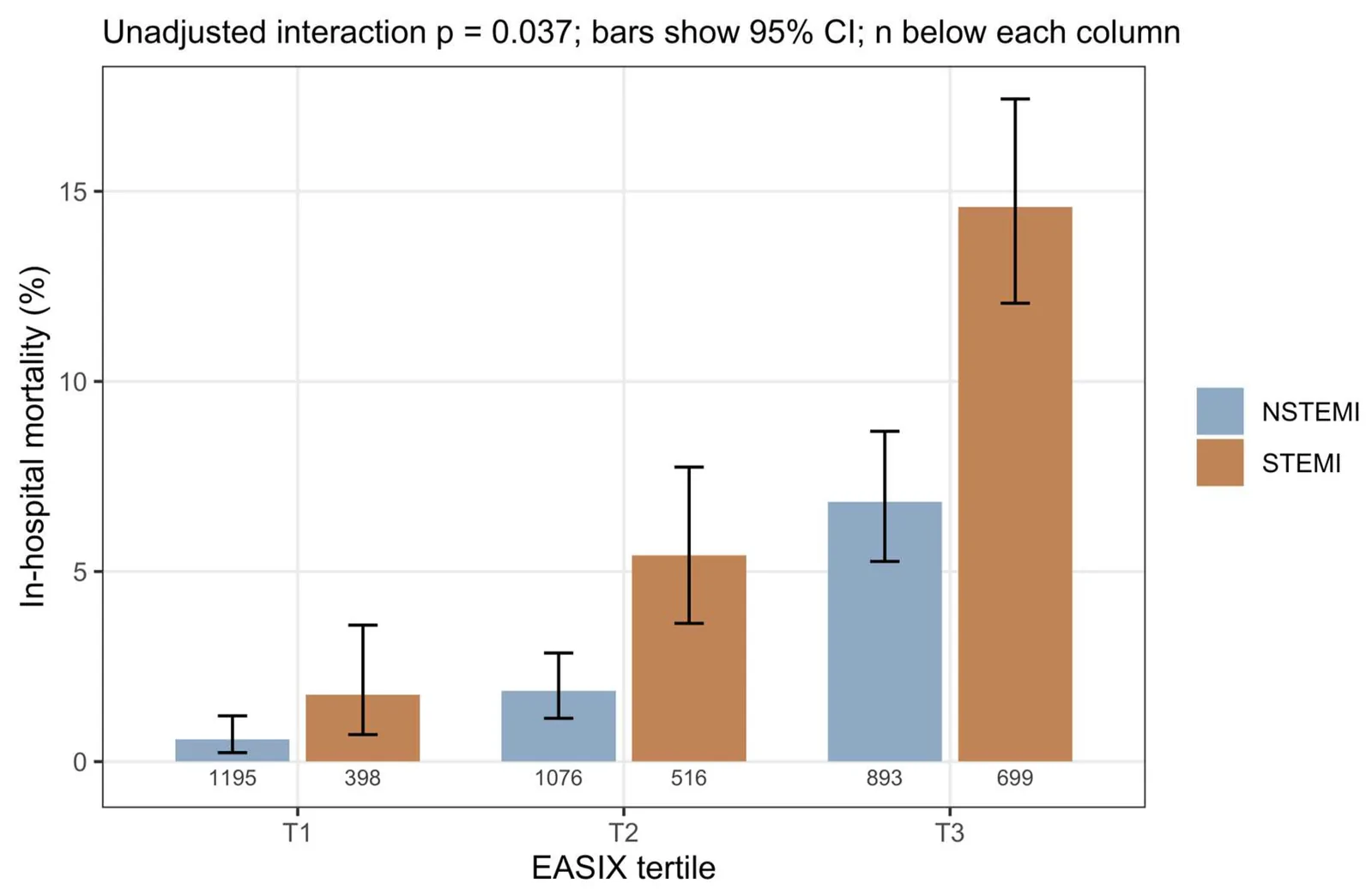
In-hospital mortality by EASIX tertile and infarct type. Bars are observed in-hospital mortality with 95% binomial confidence intervals on the index-based population (*n* = 4777, 225 deaths); the number of patients contributing to each bar is printed beneath it. Tertile cutpoints were derived in this cohort (T1 < 0.94, T2 0.94 to 1.59, T3 > 1.59) and are exploratory. The *p* value is for the unadjusted interaction between log2(EASIX) and infarct type; after adjustment for the GRACE score it was 0.278 (Supplementary Table S4). NSTEMI, non-ST-elevation myocardial infarction; STEMI, ST-elevation myocardial infarction.

The association was present in both infarct types (Supplementary Table S4). The unadjusted odds ratio per doubling was 3.488 (2.790 to 4.362) in non-ST-elevation infarction (3164 patients, 88 deaths; AUC 0.788) and 2.572 (2.149 to 3.079) in ST-elevation infarction (1613 patients, 137 deaths; AUC 0.749), with an unadjusted interaction *p* = 0.037 that was not supported after adjustment for the GRACE score (*p* = 0.278). Within the same EASIX tertile, mortality was consistently higher in ST-elevation infarction (Supplementary Table S5, Figure 3). Fitted separately within each infarct type, the GRACE-adjusted odds ratio was 2.776 (2.175 to 3.542) in NSTEMI and 2.229 (1.830 to 2.715) in STEMI, and adding EASIX raised the AUC from 0.855 to 0.894 and from 0.825 to 0.866, with optimism-corrected increments of 0.037 (0.020 to 0.062) and 0.039 (0.025 to 0.063) (Supplementary Table S6).

The GRACE-adjusted estimate was robust to the outcome definition, to index-admission management and to measures of infarct burden (Supplementary Table S7). Excluding the 13 deaths after transfer to cardiac surgery, the odds ratio was 2.504 (2.143 to 2.927) and the corrected AUC increment 0.038 (0.026 to 0.053); excluding the 577 patients referred to the heart team or to CABG, they were 2.533 (2.150 to 2.984) and 0.039 (0.027 to 0.057). Assigning ST-deviation points to every NSTEMI patient gave 2.640 (2.270 to 3.071) and 0.051 (0.037 to 0.070). Adding the revascularisation strategy, and then the final TIMI flow grade, left the odds ratio at 2.423 (2.081 to 2.822) and 2.404 (2.064 to 2.801). In the 4761 patients with a troponin value, adding log2 high-sensitivity troponin T changed the odds ratio from 2.505 (2.147 to 2.923) to 2.587 (2.195 to 3.049); in the 3416 patients with a recorded LVEF, adding LVEF changed it from 2.088 (1.691 to 2.578) to 1.897 (1.527 to 2.357).

Over the GRACE score, LDH alone added most to discrimination (increase in AUC 0.027), creatinine alone 0.014 and platelet count alone 0.002; LDH with creatinine added 0.034, and all three components entered separately 0.037, against 0.036 for EASIX entered as a single term (Supplementary Table S8). Removing any one component from the three-component model worsened fit (likelihood ratio chi-square 84.7 for LDH, 32.9 for creatinine and 16.7 for platelet count, each *p* < 0.001). EASIX correlated with high-sensitivity troponin T with a Spearman coefficient of 0.344, and LDH with troponin at 0.431. The optimism-corrected difference in AUC between the freely weighted index and EASIX was 0.007.

The distribution of each GRACE component is given in Supplementary Table S9. Age contributed a median of 58 points, ST-deviation points were assigned in 33.8% of patients and elevated cardiac markers in 96.6%.

## 4. Discussion

In 4777 consecutive patients with AMI undergoing coronary angiography, admission EASIX was independently associated with in-hospital mortality, with an approximately 2.4-fold increase in the odds of death per doubling of the index after adjustment for the GRACE score, modelled as a spline and after clinical adjustment (Table 2). Adding EASIX to a logistic model containing the GRACE score improved discrimination, the Brier score and net benefit (Table 3, Figure 2), raising the AUC from 0.855 to 0.890 with an optimism-corrected increment of 0.034. The gain was also present against the published GRACE risk after recalibration of its intercept and slope, with an optimism-corrected increment of 0.024 (Table 5), in each infarct type fitted separately, and in sensitivity analyses for the outcome definition, for index-admission management and for infarct burden. Freely estimated component weights differed from those implicit in EASIX, but the freely weighted index discriminated only slightly better.

These findings extend previous cardiovascular reports to a population defined by AMI managed invasively, rather than by intensive-care admission [7,8,13] or by a single infarct type [9,10,11,12]. The direction and approximate magnitude are consistent with the published literature. In a cohort undergoing cardiac catheterisation, log2 EASIX was associated with five-year survival with a hazard ratio of 1.29 in the training set [6]. In critically ill patients with coronary heart disease, the highest EASIX tertile carried an adjusted hazard ratio above two for both 30-day and one-year mortality [7], and a comparable gradient was reported for 30-day mortality in an intensive-care cohort with AMI [8]. In non-ST-elevation myocardial infarction, log2 EASIX was associated with one-year mortality with an adjusted hazard ratio of 1.381 in a clinical model and 1.315 after adjustment for the GRACE score [12], the latter being the closer comparator for the present analysis. Our adjusted estimate per doubling is larger than these, which is expected for an in-hospital rather than a one-year or five-year horizon, since a marker of acute physiological derangement should weigh most heavily on the earliest events. Differences in effect measure, odds ratios here against hazard ratios in those studies, and in covariate sets further limit direct numerical comparison.

More recently, Zhao et al. evaluated early EASIX in a hospital-based AMI cohort and an independent intensive-care cohort. In their primary cohort, EASIX remained associated with 30-day mortality after adjustment for GRACE and sex, and increased the AUC by 0.011, with little change in prediction error or decision curve net benefit [15]. Direct numerical comparison with the present results is limited by differences in outcome horizon and GRACE implementation: Zhao et al. evaluated 30-day mortality using a refitted GRACE-and-sex model, whereas the present study evaluated in-hospital mortality against both a refitted model and the recalibrated published GRACE 1.0 in-hospital risk.

The comparison with reports in ST-elevation infarction is less direct. Two studies of primary percutaneous coronary intervention reported associations with the no-reflow phenomenon [10,11], an endpoint we could not evaluate validly, and one reported an adjusted odds ratio of 18.97 per raw EASIX unit [11], an estimate on an untransformed scale that is not comparable with a per-doubling estimate. A third reported an adjusted odds ratio of 2.467 for contrast-induced nephropathy [9]. Our secondary estimate for CA-AKI was weaker on the same scale: an AUC of 0.645 against 0.785 (0.738 to 0.832) in that study [9]. The cohorts and the outcome definitions differ, and the structural coupling described below applies to both estimates, so we regard the comparison as uninformative rather than as evidence against the index.

The present study differs from the existing literature in two respects. First, it applies the original published GRACE in-hospital risk, evaluates its calibration in this cohort, recalibrates its intercept and slope, and then quantifies the incremental contribution of EASIX, reporting discrimination, calibration, Brier score, likelihood ratio testing, IDI and decision-analytic performance together; it also evaluates the association separately within NSTEMI and STEMI. Second, it directly tests the fixed component weights of EASIX and compares the composite with each component, component pairs and a freely weighted model. The published GRACE risk discriminated well and over-predicted modestly, and EASIX added to it after recalibration. The clinical reading is narrow but concrete. The stratification is not uniform across the risk range: among patients in the guideline high-risk category, mortality rose from 2.3% in the lowest to 16.0% in the highest EASIX tertile, and in the intermediate category every death occurred in the highest tertile, whereas in the low-risk category, where deaths were rare, the index added nothing discernible (Table 4). If the index has a use, it is in resolving risk among patients a guideline score has already placed at intermediate or high risk. The decisions such a resolution would have to be tested against are concrete enough to name, among them the intensity of monitoring, admission to coronary care rather than a step-down bed, and the timing of angiography in non-ST-elevation presentations. This analysis cannot settle any of them, and no cutpoint is proposed. The decision curve should be read against these candidate actions: its threshold range corresponds to the harm–benefit trade-offs plausible for them, and a formal evaluation would require specifying one action, its harms and its costs prospectively. Reporting calibration alongside discrimination is a requirement of contemporary prediction-model reporting standards [17], and its uneven reporting across the existing EASIX literature limits what can be concluded about clinical usefulness from those reports taken together.

On the weighting question, EASIX carries the weights of the haematological setting in which it was derived [3]. In this cohort creatinine warranted approximately 1.6 times, and platelet count approximately 0.6 times, the weight the index assigns them, and the fixed-weight model was formally rejected (Supplementary Table S3). That creatinine should carry the greater weight in an infarct population is unsurprising, since renal impairment is among the strongest determinants of mortality after an acute coronary syndrome [23]. The practical consequence was small: the optimism-corrected gain in the AUC was 0.007. The reasonable interpretation is that EASIX remains practically usable despite weights that are not optimal for this setting, not that its original weights are supported. Any freely weighted index would require derivation and validation in separate cohorts before it could be preferred.

The two infarct types differ in the determinants of early death. Patients with non-ST-elevation infarction are older and carry a heavier burden of diabetes, heart failure and previous infarction [24], whereas early death after ST-elevation infarction is driven disproportionately by mechanical complications and infarct size: in a series of 6228 infarct patients treated with percutaneous coronary intervention, mechanical complications accounted for 6.7% of index-infarct-related deaths within 6 months after ST-elevation infarction against 0.8% after non-ST-elevation infarction [25], and infarct size independently predicts mortality after anterior ST-elevation infarction [26]. Consistent with this, absolute mortality within any given EASIX tertile was higher in ST-elevation infarction (Supplementary Table S5, Figure 3). The relative association with EASIX, however, did not differ detectably between the types once the GRACE score was accounted for, and the incremental value was similar when each type was modelled separately (Supplementary Table S6).

A related question is what the composite adds to its parts. LDH carried most of the incremental information, creatinine added to it, and platelet count, although it discriminated poorly on its own, made a small but independent contribution once the other two components were known; each component was retained when the other two were in the model, and EASIX entered as a single term captured nearly all of the information in the three components entered separately (Supplementary Table S8). The increment is therefore not reducible to the re-entry of continuous creatinine into a score that carries it only in categorised form. Because LDH also rises with myocardial necrosis, part of its signal could reflect infarct size; adjustment for high-sensitivity troponin T, however, did not attenuate the association, and adjustment for LVEF attenuated it only modestly (Supplementary Table S7). The index is best read as a marker of systemic physiological stress across organ systems in the acute phase rather than as a specific measure of endothelial activation. These comparisons are internal to the index rather than independent biological evidence, and they do not establish that the multiplicative form is the best way to combine the three measurements.

The secondary analysis of CA-AKI requires particular caution. EASIX contains admission creatinine and the outcome is defined by a rise from that same value, so the index enters this comparison with a structural advantage. A reported association is therefore not independent evidence of an endothelial mechanism, and we regard this analysis as descriptive. Ascertainment is also incomplete: the outcome definition allows a creatinine rise within seven days, but the median length of stay was 2.23 days and 91.1% of survivors were discharged before day 7, so creatinine trajectories after early discharge were not observed and late CA-AKI is under-ascertained. For the same reason the maximum in-hospital creatinine, from which the outcome is derived, was excluded from every model.

Two further design choices deserve comment because they affect what this cohort can show. First, we did not exclude patients who were intubated, received inotropes or required dialysis. These are consequences of deterioration rather than admission characteristics, and conditioning on them would remove precisely the patients in whom the outcome occurs. Second, patients with advanced chronic kidney disease were not eligible. Since creatinine is a component of the index, the results should not be extrapolated to them.

### Limitations

This is a retrospective, single-centre study, and the observed event rates are those of this cohort rather than population estimates. The published GRACE risk was applied through its original points-to-probability nomogram; the later GRACE 2.0 and 3.0 models were not evaluated. ST-segment depression was not recorded, so the GRACE score understates risk in NSTEMI patients with ST depression; the upper-bound scenario did not change the conclusions. Entry required survival to coronary angiography, so the earliest and most severe deaths are structurally absent and the association applies to patients who survived to catheterisation. Findings require confirmation in other settings.

The statistical analysis plan was frozen after the analyses had been run: no result is prespecified, and all findings, including the subgroup, interaction, tertile and re-weighting analyses, are exploratory and uncorrected for multiplicity.

Several sources of unmeasured and residual confounding remain. Arrhythmia burden, the angiographic extent of coronary disease, smoking status, admission medications and the timing of reperfusion were unavailable; revascularisation strategy and final TIMI flow were available but are post-admission variables, and adding them did not change the estimate. Systolic blood pressure was available only to the nearest 5 mmHg and was not modelled continuously. Source records for the patients excluded for advanced kidney disease lacked the admission variables required for the GRACE score (Killip class, heart rate and systolic blood pressure), so a sensitivity analysis including them was not possible. The results should therefore not be extrapolated to patients with advanced kidney disease.

What the index measures is not settled. Its association with the endothelium rests on correlations with soluble markers in haematological cohorts [5]; in cardiac disease it has been validated against outcomes rather than against measured endothelial injury, so it is best read as a correlate of systemic physiological stress rather than a direct measure of endothelial activation. The index also inherits the properties of its assays: LDH is method-dependent, creatinine varies with calibration and traceability across laboratories, and the platforms used in this cohort were not extractable from the electronic record, so absolute index values and any cutpoint derived from them are not portable across laboratories, one reason the outcome-derived cutpoints in the existing literature span a wide range [9,10,12,13]. LVEF was recorded in 71.5% of patients and was examined only in a complete-case sensitivity analysis; residual confounding by it cannot be excluded.

Follow-up beyond hospital discharge was not systematically available, so the findings apply to the index admission only. Creatinine measurements were not time-stamped, so the timing windows of the CA-AKI definition could not be verified. The freely weighted index was derived and evaluated in the same sample; despite bootstrap validation of the fitting procedure, it has not been validated externally and its performance should be regarded as apparent.

## 5. Conclusions

In patients with AMI undergoing coronary angiography, admission EASIX was independently associated with in-hospital mortality and added discrimination and net benefit to the GRACE score, both when the score was refitted in this cohort and when the published GRACE risk was applied and recalibrated. The gain was consistent in both infarct types and remained present in sensitivity analyses addressing continuous creatinine, troponin and index-admission management. The component weights implicit in the index were not supported by these data, although correcting them improved discrimination only slightly. The findings are exploratory and require external validation before any clinical use.

## Figures and Tables

**Table 1:** Baseline characteristics by EASIX tertile.

Variable	T1 (<0.94) *n* = 1593	T2 (0.94–1.59) *n* = 1592	T3 (>1.59) *n* = 1592	Overall *n* = 4777	*p*
**Demographics**					
Age, years	59 (50–68)	62 (53–70)	64 (56–73)	62 (53–71)	<0.001
Male sex, *n* (%)	962 (60.4)	1146 (72.0)	1218 (76.5)	3326 (69.6)	<0.001
**Comorbidities**					
Hypertension, *n* (%)	821 (51.5)	835 (52.4)	917 (57.6)	2573 (53.9)	0.001
Diabetes mellitus, *n* (%)	573 (36.0)	525 (33.0)	600 (37.7)	1698 (35.5)	0.019
Chronic kidney disease, *n* (%)	11 (0.7)	45 (2.8)	104 (6.5)	160 (3.3)	<0.001
COPD, *n* (%)	133 (8.3)	141 (8.9)	159 (10.0)	433 (9.1)	0.257
Prior stroke, *n* (%)	61 (3.8)	76 (4.8)	106 (6.7)	243 (5.1)	0.001
Heart failure, *n* (%)	395 (24.8)	393 (24.7)	520 (32.7)	1308 (27.4)	<0.001
**Presentation**					
STEMI, *n* (%)	398 (25.0)	516 (32.4)	699 (43.9)	1613 (33.8)	<0.001
Killip class ≥ II, *n* (%)	255 (16.0)	285 (17.9)	257 (16.1)	797 (16.7)	0.278
Cardiac arrest at admission, *n* (%)	22 (1.4)	40 (2.5)	99 (6.2)	161 (3.4)	<0.001
Heart rate, bpm	104 (86–120)	103 (87–119)	104 (86–119)	104 (86–119)	0.720
Systolic blood pressure, mmHg	125 (115–135)	125 (115–135)	125 (115–135)	125 (115–135)	0.051
**Echocardiography**					
LVEF, %	60 (48–60)	55 (43–60)	50 (39–60)	53 (43–60)	<0.001
**Laboratory**					
Haemoglobin, g/dL	13.7 (12.3–14.8)	14.0 (12.6–15.2)	13.9 (12.3–15.2)	13.9 (12.4–15.1)	<0.001
White cell count, ×10^9^/L	10.0 (8.3–12.3)	10.2 (8.3–12.7)	11.0 (8.5–14.0)	10.3 (8.4–12.9)	<0.001
Platelet count, ×10^9^/L	283 (240–332)	242 (205–283)	215 (174–256)	246 (204–294)	<0.001
Creatinine, mg/dL	0.79 (0.69–0.91)	0.92 (0.82–1.07)	1.08 (0.91–1.31)	0.92 (0.78–1.09)	<0.001
eGFR, mL/min/1.73 m^2^	94 (82–103)	84 (67–96)	69 (50–87)	84 (65–98)	<0.001
LDH, U/L	236 (202–280)	308 (255–381)	475 (358–632)	310 (242–429)	<0.001
Albumin, g/L	42 (40–44)	42 (40–45)	41 (38–44)	42 (40–44)	<0.001
hs-Troponin T, ng/L	177 (52–600)	371 (83–1380)	1193 (176–3948)	380 (76–1724)	<0.001
NT-proBNP, pg/mL	397 (130–1419)	608 (188–2388)	1563 (404–5474)	725 (194–2868)	<0.001
LDL cholesterol, mg/dL	133 (104–159)	131 (102–157)	122 (94–150)	128 (100–156)	<0.001
CRP, mg/L	4.4 (1.9–11.1)	4.8 (1.9–12.9)	7.1 (2.6–28.8)	5.2 (2.0–15.2)	<0.001
**Risk score and indices**					
GRACE score	130 (111–150)	139 (120–159)	148 (128–169)	139 (120–160)	<0.001
EASIX	0.71 (0.58–0.83)	1.22 (1.08–1.40)	2.28 (1.87–3.18)	1.22 (0.83–1.87)	<0.001
Freely weighted index	4.97 (3.95–5.91)	8.88 (7.58–10.46)	17.58 (13.89–25.49)	8.88 (5.81–14.16)	<0.001
**Index-admission management**					
Revascularisation strategy					<0.001
Medical therapy, *n* (%)	493 (30.9)	396 (24.9)	343 (21.5)	1232 (25.8)	
PCI, *n* (%)	893 (56.1)	1004 (63.1)	1071 (67.3)	2968 (62.1)	
Heart-team decision or CABG, *n* (%)	207 (13.0)	192 (12.1)	178 (11.2)	577 (12.1)	
Final TIMI flow grade < 3, *n* (%)	51 (3.2)	75 (4.7)	95 (6.0)	221 (4.6)	<0.001
**Outcomes**					
In-hospital mortality, *n* (%)	14 (0.9)	48 (3.0)	163 (10.2)	225 (4.7)	<0.001
CA-AKI, *n* (%)	138 (8.7)	169 (10.7)	339 (21.4)	646 (13.6)	<0.001
Dialysis, *n* (%)	7 (0.4)	14 (0.9)	52 (3.3)	73 (1.5)	<0.001
Stent thrombosis, *n* (%)	6 (0.4)	10 (0.6)	6 (0.4)	22 (0.5)	0.481
Length of stay, days	1.98 (1.54–3.27)	2.25 (1.64–3.72)	2.69 (1.71–4.97)	2.23 (1.62–3.91)	<0.001

Continuous variables are median (IQR), compared with the Kruskal–Wallis test; categorical variables are *n* (%), compared with the chi-square test. Tertiles are shown for description only; the primary analysis models EASIX as a continuous variable. LVEF was recorded in 3416 patients and CA-AKI could be assessed in 4761 (no in-hospital creatinine after admission in the remainder); percentages use the available denominators. Numeric hs-troponin T values were retained for 4761 patients; documented positive cardiac-marker status was available for the remaining 16 patients. Systolic blood pressure was recorded to the nearest 5 mmHg. Smoking status and admission medications were not recorded. Abbreviations: CA-AKI, contrast-associated acute kidney injury; CABG, coronary artery bypass grafting; COPD, chronic obstructive pulmonary disease; CRP, C-reactive protein; EASIX, Endothelial Activation and Stress Index; eGFR, estimated glomerular filtration rate; GRACE, Global Registry of Acute Coronary Events; hs, high-sensitivity; IQR, interquartile range; LDH, lactate dehydrogenase; LDL, low-density lipoprotein; LVEF, left ventricular ejection fraction; NT-proBNP, N-terminal pro-B-type natriuretic peptide; PCI, percutaneous coronary intervention; STEMI, ST-elevation myocardial infarction; TIMI, thrombolysis in myocardial infarction.

**Table 2:** Logistic regression for in-hospital mortality. EASIX is modelled continuously on the log2 scale; the odds ratio is per doubling of EASIX. Abbreviations: CI, confidence interval; OR, odds ratio.

Model	Variable	OR (95% CI)	*p*
Model 1 (unadjusted) (*n* = 4777, 225 deaths)	log2(EASIX)	3.146 (2.738–3.614)	<0.001
Model 2 (GRACE-adjusted, primary) (*n* = 4777, 225 deaths)	log2(EASIX)	2.386 (2.053–2.774)	<0.001
	GRACE score, restricted cubic spline with four degrees of freedom	LR chi-square 251.9 on 4 df	<0.001
Model 2a (GRACE as a linear term) (*n* = 4777, 225 deaths)	log2(EASIX)	2.554 (2.202–2.963)	<0.001
	GRACE score	1.024 (1.020–1.028)	<0.001
Model 3 (clinical) (*n* = 4777, 225 deaths)	log2(EASIX)	2.363 (1.974–2.828)	<0.001
	Age	1.018 (1.002–1.034)	0.027
	Male sex	0.621 (0.439–0.878)	0.007
	Killip class	3.447 (2.945–4.034)	<0.001
	STEMI	2.217 (1.595–3.083)	<0.001
	eGFR	0.979 (0.970–0.989)	<0.001

Every model is fitted on all 4777 patients in the analytic cohort with 225 deaths; no model lost a record. Supplementary Table S2 collects the sensitivity analyses of the GRACE-adjusted estimate. Model 2 is the primary GRACE-adjusted model. The GRACE points scale is strongly non-linear on the log-odds scale in this cohort, so the score enters as a restricted cubic spline with four degrees of freedom (interior knots at GRACE 120, 139, 160; boundary knots 51 and 332); a spline has no single odds ratio per point, so the odds ratio column reports the likelihood ratio test for the spline block as a whole against Model 1 instead. Model 2a repeats the adjustment with the score as a linear term, the specification used in the existing literature, and is reported for comparability only. Model 3 includes only variables with complete data in the analytic cohort. The GRACE score was not entered together with its own components (Killip class, age, creatinine, systolic blood pressure, heart rate): doing so reverses the sign of the GRACE coefficient, a collinearity artefact.

**Table 3:** Discrimination, calibration and incremental value.

Model	AUC (95% CI)	Brier Score	Hosmer–Lemeshow *p*	Likelihood Ratio *p*	IDI
EASIX alone	0.786 (0.756–0.816)	0.0405	0.511	-	-
GRACE score, linear term	0.856 (0.834–0.878)	0.0425	<0.001	-	-
GRACE linear + EASIX	0.881 (0.861–0.900)	0.0386	0.003	<0.001	0.076
GRACE score, spline term	0.855 (0.833–0.877)	0.0402	0.146	-	-
GRACE spline + EASIX	0.890 (0.871–0.909)	0.0368	0.745	<0.001	0.078
Clinical model	0.914 (0.899–0.930)	0.0388	0.008	-	-
Clinical model + EASIX	0.929 (0.916–0.942)	0.0360	0.003	<0.001	0.056
**Quantity**		**Apparent**	**Optimism**	**Optimism-Corrected**	
AUC, GRACE spline		0.855	0.0001	0.855	
AUC, GRACE spline + EASIX		0.890	0.0016	0.889	
Increment in AUC		0.036	0.0016	0.034 (95% CI 0.025 to 0.052)	
Brier score, GRACE spline		0.0402	0.0001	0.0401	
Brier score, GRACE spline + EASIX		0.0368	−0.0000	0.0369	
IDI		0.078	0.0008	0.077 (95% CI 0.050 to 0.108)	

A dash denotes not applicable; the likelihood ratio test and the integrated discrimination improvement are defined only for a model compared with its own base model. All rows are logistic models refitted in this cohort. The published GRACE in-hospital-death risk function was not evaluated in this analysis, so no row describes a transported score. The GRACE points scale is strongly non-linear on the log-odds scale here (spline versus linear term, *p* < 0.001), so the spline specification is primary and the linear term is retained for comparison with the existing literature. DeLong test: adding EASIX to GRACE (spline), *p* < 0.001; to GRACE (linear term), *p* = 0.009; to the clinical model, *p* = 0.002. Akaike information criterion, GRACE plus EASIX: 1293.7 with the spline and 1360.3 with the linear term. The DeLong test is known to be underpowered for detecting the effect of adding a variable to an existing model, and the change in AUC alone is a weak measure of incremental value; the likelihood ratio test, IDI and decision curve should be read together with it. Bootstrap internal validation (500 resamples, seed 20260907). The base GRACE spline model and the GRACE plus EASIX model were validated together on identical resamples; knot placement and the choice between linear and spline entry of the GRACE score were repeated within each resample (the spline was preferred by the Akaike information criterion in 500 of 500 resamples). Confidence intervals are bootstrap percentile intervals. Bootstrap calibration slope: 0.977 for the GRACE spline model and 0.976 for GRACE plus EASIX; calibration intercept −0.008 and −0.008 (median across resamples). Optimism-corrected net benefit at threshold probabilities of 5%, 10%, 15% and 20%: GRACE plus EASIX 0.0278, 0.0208, 0.0126 and 0.0094; GRACE alone 0.0246, 0.0171, 0.0104 and 0.0040. Abbreviations: AUC, area under the receiver operating characteristic curve; CI, confidence interval; IDI, integrated discrimination improvement.

**Table 4:** In-hospital mortality by guideline GRACE risk category and EASIX tertile.

GRACE Category	*n* (%)	T1	T2	T3	All
Low (≤108)	723 (15.1)	0.3% (1/349)	0.0% (0/228)	0.0% (0/146)	0.1% (1/723)
Intermediate (109–140)	1786 (37.4)	0.0% (0/673)	0.0% (0/619)	2.2% (11/494)	0.6% (11/1786)
High (>140)	2268 (47.5)	2.3% (13/571)	6.4% (48/745)	16.0% (152/952)	9.4% (213/2268)
**All**	4777 (100.0)	0.9% (14/1593)	3.0% (48/1592)	10.2% (163/1592)	4.7% (225/4777)

Cells are observed in-hospital mortality with deaths over patients in the analytic cohort (*n* = 4777). Categories are the guideline in-hospital-death categories of the GRACE score. EASIX tertile cutpoints were derived in this cohort and are descriptive. Abbreviations: EASIX, Endothelial Activation and Stress Index; GRACE, Global Registry of Acute Coronary Events.

**Table 5:** Published GRACE in-hospital risk: calibration in this cohort, recalibration and incremental value of EASIX.

(**A**) **Published probabilities applied without refitting.**								
**Population**	***n* (Deaths)**	**Observed Mortality, %**	**Mean Predicted, %**	**Observed-to-Expected Ratio**	**Calibration-in-the-Large**	**Calibration Slope**	**AUC (95% CI)**	**Brier Score**
All patients	4777 (225)	4.7	5.4	0.86	−0.188	0.852	0.856 (0.834–0.878)	0.0440
NSTEMI	3164 (88)	2.8	3.5	0.80	−0.261	0.895	0.847 (0.810–0.883)	0.0282
STEMI	1613 (137)	8.5	9.3	0.91	−0.126	0.764	0.828 (0.796–0.860)	0.0751
All patients, ST-deviation points assigned to every NSTEMI	4777 (225)	4.7	8.2	0.58	−0.693	0.854	0.833 (0.808–0.857)	0.0468
(**B**) **Published linear predictor recalibrated (intercept and slope), with and without EASIX.**								
**Population**	***n* (Deaths)**	**EASIX OR per Doubling (95% CI)**	**AUC, Base Model**	**AUC + EASIX**	**Optimism-Corrected Change in AUC (95% CI)**		**LRT *p***	**IDI**
All patients	4777 (225)	2.554 (2.202–2.963)	0.856	0.881	0.024 (0.012–0.040)		<0.001	0.076
NSTEMI	3164 (88)	2.900 (2.297–3.662)	0.847	0.874	0.025 (0.004–0.059)		<0.001	0.076
STEMI	1613 (137)	2.297 (1.893–2.787)	0.828	0.852	0.023 (0.003–0.046)		<0.001	0.075
All patients, ST-deviation points assigned to every NSTEMI	4777 (225)	2.717 (2.345–3.149)	0.833	0.871	0.038 (0.024–0.058)		<0.001	0.087

Published probabilities were obtained from the points-to-probability table of the GRACE in-hospital-death nomogram, which is linear on the logit scale: logit(p) = −8.0922 + 0.03271 × GRACE points. Recalibration re-estimates only an intercept and a slope, so the ranking of patients, and hence the AUC of the base model, is that of the published score. Optimism-corrected changes use 500 bootstrap resamples in which both models were refitted. ST-segment depression was not recorded; the last row of each panel assigns ST-deviation points to every NSTEMI patient as an upper bound. Abbreviations: AUC, area under the receiver operating characteristic curve; CI, confidence interval; EASIX, Endothelial Activation and Stress Index; GRACE, Global Registry of Acute Coronary Events; IDI, integrated discrimination improvement; LRT, likelihood ratio test; OR, odds ratio. NSTEMI, non-ST-elevation myocardial infarction; STEMI, ST-elevation myocardial infarction.

## Data Availability

The data presented in this study are available on request from the corresponding author. The data are not publicly available because they contain information that could compromise the privacy of research participants.

## Supplementary Materials

The following supporting information can be downloaded at: https://www.mdpi.com/article/10.3390/jcm15197703/s1, Table S1: In-hospital mortality and unadjusted odds ratios by EASIX tertile; Table S2: Sensitivity analyses of the GRACE-adjusted estimate; Table S3: Re-derivation of the EASIX component weights; Table S4: Subgroup analysis by infarct type and interaction; Table S5: In-hospital mortality within the same EASIX tertile, by infarct type; Table S6: Separate modelling by infarct type; Table S7: Sensitivity analyses for outcome definition, index-admission management and infarct burden; Table S8: Contribution of the individual EASIX components beyond the GRACE score; Table S9: Distribution of the GRACE score components.
