# Supplementary material for: Admission Endothelial Activation and Stress Index in Acute Myocardial Infarction: Incremental Value Beyond the GRACE Score and a Test of the Index Weights
Source: J Clin Med. 2026 Oct 5;15(19):7703. doi: 10.3390/jcm15197703 (PMC13666320; doi:10.3390/jcm15197703)
Supplement: Supplementary file 1 [file jcm-15-07703-s001.zip › jcm-4584285-supplementary.pdf]

Journal of
Clinical Medicine

MDPI

Supplementary Materials

Admission Endothelial Activation and Stress Index in Acute Myocardial Infarction: Incremental Value beyond the GRACE Score and a Test of the Index Weights

Şahin K.E., Duman A.B., Dalgıç Ş.N., Karaçimen D., Dalgıç Y., Çağdaş M., Avcı S. and Hoşoğlu A.

Supplementary Table S1. In-hospital mortality and unadjusted odds ratios by EASIX tertile.

Tertile	n	Deaths n (%)	Unadjusted OR (95% CI)
T1	1593	14 (0.88)	1 (reference)
T2	1592	48 (3.02)	3.51 (1.93–6.39)
T3	1592	163 (10.24)	12.86 (7.42–22.31)

p for trend <0.001. Categorisation loses information (AUC 0.786 versus 0.732), so tertiles are presented for description only.

Supplementary Table S2. Sensitivity analyses of the GRACE-adjusted estimate.

Analysis	Population	log2(EASIX) OR (95% CI)	*p*
Model 2, primary (GRACE spline)	4777 (225 deaths)	2.386 (2.053–2.774)	<0.001
Model 2a, GRACE as a linear term	4777 (225 deaths)	2.554 (2.202–2.963)	<0.001
After 1% winsorisation of all three components	4777 (225 deaths)	2.466 (2.104–2.891)	<0.001
Substituting the freely weighted index	4777 (225 deaths)	2.300 (1.993–2.654)	<0.001

All rows except Model 2a adjust for the GRACE score as a restricted cubic spline with four degrees of freedom, the primary specification; Model 2a enters the score as a linear term and is shown for comparability with the existing literature. The area under the curve for the winsorised index was 0.786 (0.756–0.816), against 0.786 (0.756–0.816) for the index as recorded. Every row is fitted on all 4777 patients in the analytic cohort.

Supplementary Table S3. Re-derivation of the EASIX component weights (exploratory).

Component	Weight in Classical EASIX	Re-Derived Weight	Bootstrap 95% CI	Classical Value Outside the CI
log2(LDH)	1.000	1.000	reference	-
log2(Creatinine)	1.000	1.605	1.213 to 2.046	**Yes**
log2(Platelet count)	−1.000	−0.626	−0.935 to −0.333	**Yes**

Confidence intervals are percentile intervals from 500 bootstrap resamples.

Likelihood ratio test (freely weighted versus fixed weights): chi-square = 22.63, df = 2, $p < 0.001$. AIC 1537.5 versus 1518.9.

Index	Formula	AUC (95% CI)
Classical EASIX	LDH × creatinine/platelets	0.786 (0.756–0.816)
Freely weighted index	LDH × creatinine$^{1.605}$/platelets$^{0.626}$	0.795 (0.765–0.825)

DeLong p = 0.074.

Interpretation. The fixed weights are statistically rejected: both bootstrap confidence intervals exclude the classical value. Correcting them improves discrimination only slightly (optimism-corrected difference in Supplementary Table S8). The weights were derived in the same sample, no external validation was performed, and the finding is exploratory.

Supplementary Table S4. Subgroup analysis by infarct type and interaction (exploratory).

Subgroup	n	Deaths n (%)	EASIX Median (IQR)	AUC (95% CI)	OR (95% CI)	*p*
NSTEMI	3164	88 (2.78)	1.13 (0.79–1.69)	0.788 (0.740–0.836)	3.488 (2.790–4.362)	<0.001
STEMI	1613	137 (8.49)	1.44 (0.95–2.31)	0.749 (0.706–0.792)	2.572 (2.149–3.079)	<0.001

Interaction test (log2(EASIX) x infarct type): *p* = 0.037. Counts are on the analytic cohort (n = 4777, 225 deaths). The interaction p above is unadjusted; after adjustment for the GRACE score (restricted cubic spline) it was 0.278. The unadjusted odds ratio was larger in NSTEMI, but the difference was not supported after GRACE adjustment. These analyses are exploratory and no correction for multiplicity was applied. Abbreviations: AUC, area under the receiver operating characteristic curve; CI, confidence interval; IQR, interquartile range; NSTEMI, non-ST-elevation myocardial infarction; OR, odds ratio; STEMI, ST-elevation myocardial infarction.

Supplementary Table S5. In-hospital mortality within the same EASIX tertile, by infarct type.

Infarct Type	T1	T2	T3
NSTEMI	0.6%	1.9%	6.8%
STEMI	1.8%	5.4%	14.6%

Within the same EASIX tertile, mortality differs substantially by infarct type; a pooled tertile presentation conceals this difference.

Supplementary Table S6. Separate modelling by infarct type.

Population	n (Deaths)	EASIX OR per Doubling (95% CI)	AUC, Base Model	AUC, + EASIX	Optimism-corrected Change in AUC (95% CI)	LRT p	IDI
NSTEMI	3164 (88)	2.776 (2.175–3.542)	0.855	0.894	0.037 (0.020–0.062)	<0.001	0.086
STEMI	1613 (137)	2.229 (1.830–2.715)	0.825	0.866	0.039 (0.025–0.063)	<0.001	0.079

Infarct type	Unadjusted OR (95% CI)	GRACE spline-adjusted OR (95% CI)	GRACE linear-adjusted OR (95% CI)	Clinically adjusted OR (95% CI)
NSTEMI	3.488 (2.790–4.362)	2.776 (2.175–3.542)	2.900 (2.297–3.662)	2.757 (2.084–3.647)
STEMI	2.572 (2.149–3.079)	2.229 (1.830–2.715)	2.297 (1.893–2.787)	2.109 (1.671–2.663)

The base model is GRACE as a restricted cubic spline with four degrees of freedom, refitted within each infarct type. Clinical adjustment: age, sex, Killip class and eGFR. Interaction between log2(EASIX) and infarct type in the GRACE-adjusted model: p 0.278. Odds ratios are per doubling

of EASIX. Abbreviations: AUC, area under the receiver operating characteristic curve; CI, confidence interval; EASIX, Endothelial Activation and Stress Index; GRACE, Global Registry of Acute Coronary Events; IDI, integrated discrimination improvement; LRT, likelihood ratio test; OR, odds ratio. eGFR, estimated glomerular filtration rate; NSTEMI, non-ST-elevation myocardial infarction; STEMI, ST-elevation myocardial infarction.

Supplementary Table S7. Sensitivity analyses for outcome definition, index-admission management and infarct burden.

**A. Outcome definition and ST-deviation scoring**							
**Population**	**n (Deaths)**	**EASIX OR per Doubling (95% CI)**	**AUC, Base Model**	**AUC, + EASIX**	**Optimism-corrected Change in AUC (95% CI)**	**LRT p**	**IDI**
All in-hospital deaths (primary)	4777 (225)	2.386 (2.053–2.774)	0.855	0.890	0.034 (0.025–0.052)	<0.001	0.078
Deaths after transfer to cardiac surgery excluded	4764 (212)	2.504 (2.143–2.927)	0.859	0.898	0.038 (0.026–0.053)	<0.001	0.084
Patients referred to the heart team or CABG excluded	4200 (195)	2.533 (2.150–2.984)	0.862	0.902	0.039 (0.027–0.057)	<0.001	0.085
ST-deviation points assigned to every NSTEMI	4777 (225)	2.640 (2.270–3.071)	0.831	0.883	0.051 (0.037–0.070)	<0.001	0.092

**B. Index-admission management and infarct burden**			
Model	n (deaths)	EASIX OR per doubling (95% CI)	AUC (95% CI)
GRACE + EASIX	4777 (225)	2.386 (2.053–2.774)	0.890 (0.871–0.909)
GRACE + EASIX + revascularisation strategy	4777 (225)	2.423 (2.081–2.822)	0.893 (0.875–0.911)
GRACE + EASIX + strategy + final TIMI flow <3	4777 (225)	2.404 (2.064–2.801)	0.894 (0.876–0.912)
GRACE + EASIX, patients with hs-TnT	4761 (213)	2.505 (2.147–2.923)	0.890 (0.870–0.910)
GRACE + EASIX + log2 hs-TnT	4761 (213)	2.587 (2.195–3.049)	0.891 (0.871–0.910)
GRACE + EASIX, patients with LVEF	3416 (96)	2.088 (1.691–2.578)	0.846 (0.813–0.879)
GRACE + EASIX + LVEF	3416 (96)	1.897 (1.527–2.357)	0.860 (0.828–0.891)

GRACE enters as a restricted cubic spline with four degrees of freedom. Revascularisation strategy (medical therapy, PCI, heart-team decision or CABG) and final TIMI flow are post-admission variables and are shown as sensitivity analyses only. hs-TnT and LVEF models are complete-case (hs-TnT available in 4761, LVEF in 3416 patients). Abbreviations: AUC, area under the receiver operating characteristic curve; CI, confidence interval; EASIX, Endothelial Activation and Stress Index; GRACE, Global Registry of Acute Coronary Events; IDI, integrated discrimination improvement; LRT, likelihood ratio test; OR, odds ratio. CABG, coronary artery bypass grafting; hs-TnT, high-sensitivity troponin T; LVEF, left ventricular ejection fraction; NSTEMI, non-ST-elevation myocardial infarction; PCI, percutaneous coronary intervention; TIMI, Thrombolysis in Myocardial Infarction.

Supplementary Table S8. Contribution of the individual EASIX components beyond the GRACE score.

**Model**	**Added df**	**LR Chi-Square vs GRACE**	***p***	**AUC (95% CI)**	**Change in AUC**	**Brier Score**	**AIC**
GRACE	-	-	-	0.855 (0.833–0.877)	-	0.0402	1429.8
GRACE + log2 LDH	1	91.5	<0.001	0.882 (0.862–0.901)	0.027	0.0383	1340.3
GRACE + log2 creatinine	1	47.7	<0.001	0.869 (0.848–0.890)	0.014	0.0392	1384.1
GRACE + log2 platelet count	1	12.4	<0.001	0.856 (0.834–0.879)	0.002	0.0397	1419.4
GRACE + log2 LDH + log2 creatinine	2	126.4	<0.001	0.889 (0.870–0.908)	0.034	0.0373	1307.5
GRACE + log2 LDH + log2 platelet count	2	110.1	<0.001	0.885 (0.865–0.904)	0.030	0.0376	1323.7
GRACE + log2 creatinine + log2 platelet count	2	58.3	<0.001	0.871 (0.850–0.892)	0.016	0.0388	1375.5
GRACE + all three components	3	143.1	<0.001	0.892 (0.873–0.911)	0.037	0.0368	1292.8
GRACE + EASIX	1	138.2	<0.001	0.890 (0.871–0.909)	0.036	0.0368	1293.7

Component removed from the three-component model	LR chi-square (1 df)	p
log2 LDH	84.7	<0.001
log2 creatinine	32.9	<0.001
log2 platelet count	16.7	<0.001

	log2 EASIX	log2 LDH	log2 creatinine	log2 platelet count	log2 hs-TnT
log2 EASIX	1.000	0.723	0.550	−0.451	0.344
log2 LDH	0.723	1.000	0.136	0.043	0.431
log2 creatinine	0.550	0.136	1.000	−0.076	0.122
log2 platelets	−0.451	0.043	−0.076	1.000	0.040
log2 hs-TnT	0.344	0.431	0.122	0.040	1.000

GRACE enters every model as a restricted cubic spline with four degrees of freedom; components are on the log2 scale. The three-component model is the freely weighted composite refitted alongside the GRACE score. The correlation matrix gives Spearman coefficients (n = 4761). Freely weighted index versus EASIX, both without GRACE: apparent difference in AUC 0.009, optimism 0.0018, optimism-corrected difference 0.007 (500 bootstrap resamples with the weights re-derived in each). Abbreviations: AUC, area under the receiver operating characteristic curve; CI, confidence interval; EASIX, Endothelial Activation and Stress Index; GRACE, Global Registry of Acute Coronary Events; IDI, integrated discrimination improvement; LRT, likelihood ratio test; OR, odds ratio. AIC, Akaike information criterion; df, degrees of freedom; hs-TnT, high-sensitivity troponin T; LDH, lactate dehydrogenase.

Supplementary Table S9. Distribution of the GRACE score components.

Component	Median (IQR) Points	Range	Patients with Non-Zero points, %
Age	58 (41–75)	0–100	99.1
Heart rate	15 (9–24)	0–38	98.1
Systolic blood pressure	34 (34–43)	10–58	100.0
Creatinine	7 (4–7)	1–21	100.0
Killip class	0 (0–0)	0–59	16.7
Cardiac arrest at admission	0 (0–0)	0–39	3.4
ST-segment deviation	0 (0–28)	0–28	33.8
Elevated cardiac markers	14 (14–14)	0–14	96.6
Total GRACE score	139 (120–160)	51–332	-

Points follow the GRACE in-hospital death nomogram and were computed from the recorded admission values (n = 4777). ST-segment depression was not recorded, so ST-deviation points are assigned to STEMI only; elevated cardiac markers were defined as admission hs-TnT above 14 ng/L. By guideline category, 15.1% scored <=108, 37.4% scored 109–140, 47.5% scored >140. Abbreviations: GRACE, Global Registry of Acute Coronary Events; hs-TnT, high-sensitivity troponin T; IQR, interquartile range; STEMI, ST-elevation myocardial infarction.
